# Mr. Alfred E. Bradley

**Published:** 1898-04

**Authors:** 


Mr. Alfred E. Bradley, a student in the medical department of Niagara University, died at the hospital of the Sisters of Charity, March 9, 1898, of typhoid fever after an illness of about ten days' duration. Mr. Bradley had displayed great aptitude for his chosen profession and his work as a student had been of a highly creditable nature. His death was a sad blow to his class.

